# The effect of an antenatal lifestyle intervention in overweight and obese women on circulating cardiometabolic and inflammatory biomarkers: secondary analyses from the LIMIT randomised trial

**DOI:** 10.1186/s12916-017-0790-z

**Published:** 2017-02-14

**Authors:** Lisa J. Moran, Louise M. Fraser, Tulika Sundernathan, Andrea R. Deussen, Jennie Louise, Lisa N. Yelland, Rosalie M. Grivell, Anne Macpherson, Matthew W. Gillman, Jeffrey S. Robinson, Julie A. Owens, Jodie M. Dodd

**Affiliations:** 1 The University of Adelaide, The Robinson Research Institute, and Discipline of Obstetrics & Gynaecology, Adelaide, South Australia Australia; 2 Monash University, Monash Centre for Health Research Implementation, Clayton, Victoria Australia; 3 The University of Adelaide, School of Population Health, Adelaide, South Australia Australia; 4 South Australian Health and Medical Research Institute, Adelaide, South Australia Australia; 5 Flinders University, Department of Obstetrics & Gynaecology, Bedford Park, South Australia Australia; 6 Obesity Prevention Program, Department of Population Medicine, Harvard Medical School, and Harvard Pilgrim Health Care Institute, Boston, Massachusetts USA; 7 Environmental Influences on Child Health Outcomes (ECHO) Program, National Institutes of Health, Rockville, Maryland USA; 8 The Women’s and Children’s Hospital, Women’s and Babies Division, Department of Perinatal Medicine, Adelaide, South Australia Australia

**Keywords:** Pregnancy, Overweight and obesity, Dietary and lifestyle intervention, Randomised trial, Cardiometabolic markers, Inflammatory markers

## Abstract

**Background:**

Maternal overweight and obesity during pregnancy is associated with insulin resistance, hyperglycaemia, hyperlipidaemia and a low-grade state of chronic inflammation. The aim of this pre-specified analysis of secondary outcome measures was to evaluate the effect of providing antenatal dietary and lifestyle advice on cardiometabolic and inflammatory biomarkers.

**Methods:**

We conducted a multicentre trial in which pregnant women who were overweight or obese were randomised to receive either Lifestyle Advice or Standard Care. We report a range of pre-specified secondary maternal and newborn cardiometabolic and inflammatory biomarker outcomes. Maternal whole venous blood was collected at trial entry (mean 14 weeks gestation; non-fasting), at 28 weeks gestation (fasting), and at 36 weeks gestation (non-fasting). Cord blood was collected after birth and prior to the delivery of the placenta. A range of cardiometabolic and inflammatory markers were analysed (total cholesterol, triglycerides, non-esterified fatty acids, high-density lipoprotein cholesterol, insulin, glucose, leptin, adiponectin, C-reactive protein, granulocyte macrophage-colony stimulating factor, interferon gamma, TNF-α, and interleukins 1β, 2, 4, 5, 6, 8, and 10). Participants were analysed in the groups to which they were randomised, and were included in the analyses if they had a measure at any time point.

**Results:**

One or more biological specimens were available from 1951 women (989 Lifestyle Advice and 962 Standard Care), with cord blood from 1174 infants (596 Lifestyle Advice and 578 Standard Care). There were no statistically significant differences in mean cardiometabolic and inflammatory marker concentrations across pregnancy and in infant cord blood between treatment groups. Estimated treatment group differences were close to zero, with 95% confidence intervals spanning a range of differences that were short of clinical relevance. There was no evidence to suggest that the intervention effect was modified by maternal BMI category.

**Conclusions:**

Despite our findings, it will be worth considering potential relationships between cardiometabolic and inflammatory markers and clinical outcomes, including longer-term infant health and adiposity.

**Trial Registration:**

Australian and New Zealand Clinical Trials Registry (ACTRN12607000161426; Date Registered 09/03/2007).

**Electronic supplementary material:**

The online version of this article (doi:10.1186/s12916-017-0790-z) contains supplementary material, which is available to authorized users.

## Background

Worldwide, more than 1.46 billion adults [1] are overweight or obese. Not only has the prevalence of obesity increased substantially over the past three decades, there appears to be no country worldwide in which this trend has been successfully averted or reversed [2]. Overweight and obesity are associated with poorer health outcomes for individuals, including hypertension, cardiovascular disease and diabetes [3].

Overweight and obesity also represent a significant health concern for women during pregnancy and childbirth, with approximately 50% of pregnant women estimated to enter pregnancy with a body mass index (BMI) above 25 kg/m^2^ [4–6]. Women who are overweight or obese during pregnancy are at increased risk of a number of complications, including gestational diabetes, hypertension and pre-eclampsia, caesarean birth, and high infant birth weight [7, 8]. In addition to pregnancy-specific risks, there is also evidence of a persisting longer-term health legacy, both for the woman and her infant. Women who are overweight or obese during pregnancy are at increased risk of developing diabetes [9], hypertension and cardiovascular disease in later life [10, 11]. Maternal obesity also identifies women who are at increased risk of premature death, largely related to major cardiovascular events [12]. Furthermore, children born to women who are overweight or obese have a significantly increased risk of both early infant and child obesity, and its subsequent complications [13–15].

Maternal obesity and gestational diabetes share a similar metabolic environment, characterised by insulin resistance, hyperglycaemia, hyperlipidaemia and a low-grade state of chronic inflammation, all of which influence the availability and transfer of nutrients to the developing fetus [16]. Importantly, maternal triglyceride concentrations correlate with infant birthweight, independent of maternal glycaemic status [17], with increased fatty acid production and placental transfer [16]. Furthermore, a strong link exists between obesity and altered glucose metabolism, and pro-inflammatory markers such as C-reactive protein (CRP), interleukin-6 (IL-6) and tumour necrosis factor alpha (TNF-α) [18–24]. Some of these molecules antagonise effects of insulin such as leptin or TNF-α, while others have beneficial effects such as adiponectin [18–24], with evidence that plasma CRP relates to insulin resistance independent of obesity [24, 25]. Pregnancy is also associated with a transient worsening of the cardiovascular risk profile including increases in dyslipidaemia, insulin resistance, oxidative stress, cytokines such as IL-6 and TNF-α, and inflammatory markers such as CRP [26–28]. These elevations in cardiovascular risk factors during pregnancy are further worsened in clinical states such as gestational diabetes, pre-eclampsia or obesity, which are associated with a poorer lipid profile, insulin resistance and elevated high-sensitivity CRP, IL-6 and TNF-α [29–33]. Overall, however, there is limited research examining changes in cardiovascular risk factors in these high risk populations, and the existing literature is generally confined to small sample sizes, and cross-sectional assessments of women across pregnancy, rather than longitudinal comparisons.

While features of this metabolic environment have been described to a limited extent among pregnant women who are overweight or obese, it is unclear whether an antenatal dietary and lifestyle intervention can change or improve the milieu. There is some evidence that physical activity during pregnancy is associated with reduced concentrations of cholesterol, triglycerides [34] and TNF-α [35], while low glycaemic index and low fat diets during pregnancy have been associated with alterations in both lipid profiles and CRP concentrations [36, 37], although such changes have not been universally reported [38]. Importantly, these studies have involved relatively lean women. The effect of antenatal dietary and lifestyle interventions on cardiometabolic and inflammatory biomarkers in overweight and obese pregnant women is uncertain, but is of particular relevance given that this group of women is considered at high risk of both pregnancy complications, including pre-eclampsia and gestational diabetes, and the future development of hypertension, cardiovascular disease and type-2 diabetes.

The primary findings of the LIMIT randomised trial evaluating the effect of an antenatal dietary and lifestyle intervention for overweight or obese pregnant women have been reported previously. Although there was no significant difference between groups in relation to the primary outcome of large-for-gestational-age infants, there was a significant 18% relative risk reduction in infant birthweight above 4 kg [39]. Likewise, despite no difference in gestational weight gain, women who received the intervention were successful in improving their diet and physical activity patterns [40]. The aim of this pre-specified analysis of secondary outcome measures was to evaluate the effect of providing antenatal dietary and lifestyle advice on maternal and infant cardiometabolic and inflammatory biomarkers.

## Methods

### Study design

We conducted a multicentre randomised trial in which women were recruited from the three major metropolitan maternity hospitals within Adelaide, South Australia. The methods [41] and primary clinical findings [39, 40, 42] of the LIMIT randomised trial have been reported in detail previously. The trial was registered on the Australian and New Zealand Clinical Trials Registry (ACTRN12607000161426).

### Inclusion and exclusion criteria

Women with a BMI ≥ 25 kg/m^2^ and singleton pregnancy between 10^+0^ and 20^+0^ weeks gestation were eligible for inclusion. Women with a multiple pregnancy, or who were diagnosed with type 1 or 2 diabetes prior to pregnancy, or who were unable to provide informed consent were ineligible to participate.

### Randomisation, masking and group allocation

The central randomisation service utilised a computer-generated randomisation schedule, with balanced variable blocks and stratification for parity (0 versus 1 or more), BMI at the time of the first antenatal appointment (25–29.9 vs. ≥ 30), and collaborating centre. Women were randomised to either ‘Lifestyle Advice’ or ‘Standard Care’ groups.

### Treatment schedules

#### Lifestyle advice

Women allocated to the Lifestyle Advice group participated in a comprehensive intervention over pregnancy, and included combined dietary, physical activity and behavioural strategies, delivered by a research dietitian and trained research assistants [41]. The dietary advice provided was consistent with current Australian standards [43]. Physical activity advice encouraged women to increase their walking and incidental activity [44]. The content of the lifestyle intervention has been described in detail previously [39, 41, 42].

#### Standard care

Women allocated to the Standard Care group continued their pregnancy care according to the guidelines of their local hospital where they had planned to birth.

### Study outcomes

All women presenting for antenatal care had their height and weight measured, and BMI calculated at the time of their first antenatal appointment, and again at 36 weeks gestation. Maternal whole venous blood was collected at the time of trial entry (mean 14 weeks gestation; non-fasting), at 28 weeks gestation (fasting), and at 36 weeks gestation (non-fasting). Cord blood was collected after birth and prior to the delivery of the placenta. After collection in lithium heparin tubes, each sample was spun in a refrigerated centrifuge, and aliquots were then stored frozen at –80 °C until analysis.

#### Cardiometabolic markers

The following cardiometabolic markers were analysed: total cholesterol, triglycerides, non-esterified fatty acids (NEFA), high-density lipoprotein cholesterol (HDL-C), insulin, glucose, leptin, adiponectin and CRP. Very low-density cholesterol (VLDL-C) was calculated as one-fifth of the triglycerides. Low-density lipoprotein cholesterol (LDL-C) was calculated using the Friedewald formula: estimated LDL = [total cholesterol] − [total HDL] − [estimated VLDL].

Glucose, cholesterol, HDL-C, triglycerides, NEFA and CRP were measured using commercial kits (Roche Diagnostics, Australia or Wako Pure Chemical Industries, Japan for NEFA) with all assays performed on the automated Hitachi Auto 912 analyser or Cobas Integra 400 Plus with appropriate calibrators and quality controls (Roche for Roche assays; and Wako standard and Sero QC’s for the NEFA C assay). Insulin concentration was analysed using highly specific radioimmuno assays (in duplicate, Linco, Millipore, MA, USA). Plasma leptin (in singulate; HL-81 K; Millipore, St. Charles, MO, USA) and plasma adiponectin (in singulate; HADP-61HK; Millipore, St. Charles, MO, USA) were determined by double antibody radioimmunoassay following the methods from the supplier.

#### Inflammatory markers

The following inflammatory markers were analysed: granulocyte macrophage-colony stimulating factor (GMCSF), interferon gamma (IFN-γ), TNF-α, and interleukins (IL) 1β, 2, 4, 5, 6, 8 and 10. Inflammatory marker concentrations were measured in plasma samples using the commercially available Invitrogen Human Ultrasensitive Cytokine Magnetic 10-Plex Panel (Life Technologies, Carlsbad, CA, USA). Assays were read using a Luminex 200 Analyser (Luminex Corporation, Austin, TX, USA) with commercially available calibrators (Life Technologies, Carlsbad, CA, USA) and an in-house quality control.

### Analysis and reporting of results

Analyses were performed on an intention to treat basis, according to the treatment group allocated at randomisation (Lifestyle Advice or Standard Care). Women were included in the analysis if they had a specimen available at one or more time points, and did not withdraw consent to use their data, or have a miscarriage, or termination of pregnancy. Maternal biomarkers (trial entry, 28 weeks, and 36 weeks gestation) were modelled separately to cord blood measures. Many of the outcomes exhibited highly skewed distributions, were log transformed prior to analysis, and are presented as median and interquartile range, with the estimate of treatment effects back-transformed to the original scale and reported as ratios of geometric means (approximately ratios of medians). Outcomes that were not log transformed are presented as means and standard deviations, and estimates of treatment effect are reported as differences in means. Statistical significance was assessed at the two-sided *P* < 0.05 level, and no adjustment was made for multiple comparisons. Analyses were performed using SAS v9.4 (Cary, NC, USA) and Stata v13 (StataCorp, Texas, USA).

Different modelling approaches were taken according to whether biomarkers were measured at multiple time points or only at one time, and according to whether there were any values above or below the assay limits of detection (tabulation of number of results outside detection limits for each biomarker can be found in Additional file 1). For biomarkers measured at only one time point, linear regression models were used, or Tobit regression models if there were values outside detection thresholds. For biomarkers measured at multiple time points, mixed models (with random effects for participant) were used to account for correlation due to repeated measures, and if there were values above or below detection thresholds, a longitudinal Tobit regression approach was adopted [45]. A time-by-treatment interaction term was included in the model to test for differences between groups in change over time. Separate estimates of treatment effect at each time point were derived, regardless of the significance of the interaction term.

Both adjusted and unadjusted analyses were performed, with adjusted analyses including stratification variables (parity, BMI category, and centre) as well as index of socioeconomic disadvantage (Socio-Economic Indexes for Areas Index of Relative Socio-Economic Disadvantage Quintile), maternal smoking status and maternal age at trial entry, as covariates. These were pre-specified adjustment variables in the main LIMIT analyses, as they were identified as potential confounders. A range of sensitivity analyses were performed, including testing the effect of different modelling assumptions, and testing the effect of fasting versus non-fasting for maternal glucose, insulin and triglycerides. Exploratory analyses were also performed to test for modification of the effect of the intervention by maternal BMI category. Estimates of treatment effect were not substantially different in these analyses.

### Sample size

The sample size of 2180 women was pre-determined, and based on the primary outcome of the trial (large for gestational age infant) as reported previously [39].

### Ethics

Approval was granted by the Women’s and Children’s Local Health Network Human Research and Ethics Committee at the Women’s and Children’s Hospital, the Central Northern Adelaide Health Service Ethics of Human Research Committee (Lyell McEwin Hospital) and the Flinders Clinical Research Ethics Committee (Flinders Medical Centre). All participants provided written informed consent to participate.

## Results

**Fig. 1:**
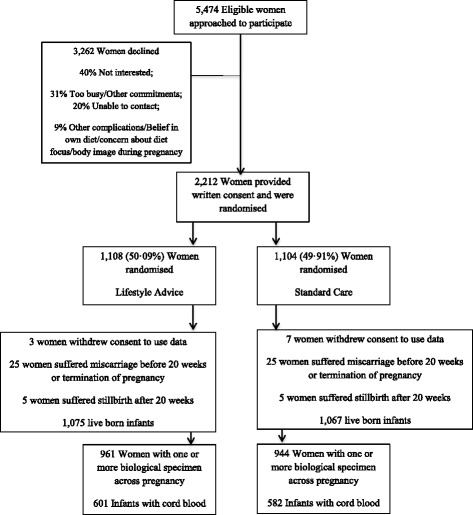
Flow of participants through the trial

Between June 2008 and December 2011, a total of 2212 women were recruited and randomised to the LIMIT trial, with 1108 allocated to receive Lifestyle Advice and 1104 to Standard Care (Fig. 1). One or more biological specimens were available from 1905 women (961 Lifestyle Advice and 944 Standard Care), of whom 1224 (64%, 634 Lifestyle Advice and 590 Standard Care) had measures at all three time points and 1622 (85%, 830 Lifestyle Advice and 792 Standard Care) had at least two measures. Cord blood was available from 1183 infants (601 Lifestyle Advice and 582 Standard Care). Detailed information on number of participants for each outcome is available in Additional file 1. There were no differences between the two treatment groups in the baseline characteristics of participants (Table 1) and women who provided a biological specimen were similar in their characteristics to the entire randomised cohort [39] (data not shown).

**Table 1:** Baseline characteristics of participants contributing one or more biological specimens

Characteristic	Lifestyle advice group *n* = 992	Standard care group *n* = 969	Total *n* = 1961
Maternal age (years), mean (SD)	29.3 (5.5)	29.6 (5.6)	29.5 (5.5)
Gestational age at entry (weeks), median (IQR)	14.0 (11.9–16.9)	14.1 (11.9–17.0)	14.1 (11.9–17.0)
Body mass index (kg/m^2^), median (IQR)	31.2 (28.1–35.9)	31.1 (27.8–35.8)	31.1 (28.0–35.9)
Body mass index category, N (%)			
25.0–29.9	409 (41.2)	408 (42.1)	817 (41.7)
30.0–34.9	290 (29.2)	286 (29.5)	576 (29.4)
35.0–39.9	184 (18.5)	159 (16.4)	343 (17.5)
≥40.0	109 (11.0)	116 (12.0)	225 (11.5)
Public patient, N (%)	974 (98.2)	944 (97.4)	1918 (97.8)
Weight (kg), mean (SD)	88.7 (17.3)	88.5 (17.6)	88.6 (17.4)
Height (m), mean (SD)	1.6 (0.1)	1.6 (0.1)	1.6 (0.1)
Race: N (%)			
Caucasian	899 (90.6)	885 (91.3)	1784 (91.0)
Asian	21 (2.1)	30 (3.1)	51 (2.6)
Other	72 (7.3)	54 (5.6)	126 (6.4)
Smoker, N (%)	142 (14.3)	116 (12.0)	258 (13.2)
Nulliparous, N (%)	407 (41.0)	389 (40.1)	796 (40.6)
Index of socio-economic disadvantage, N (%)			
Unknown	1 (0.1)	1 (0.1)	2 (0.1)
Quintile 1 (Most Disadvantaged)	308 (31.0)	284 (29.3)	592 (30.2)
Quintile 2	242 (24.4)	238 (24.6)	480 (24.5)
Quintile 3	156 (15.7)	149 (15.4)	305 (15.6)
Quintile 4	133 (13.4)	154 (15.9)	287 (14.6)
Quintile 5 (Least Disadvantaged)	152 (15.3)	143 (14.8)	295 (15.0)

### Cardiometabolic markers

Mean concentrations of cardiometabolic markers across pregnancy (Table 2) and in infant cord blood (Table 3) were similar between the two treatment groups. For cholesterol, glucose, HDL and LDL (which were not log-transformed), estimated treatment group differences were all close to zero at all time points, and 95% confidence intervals (CIs) ranged from a decrease of 0.1 to 0.2 units, to an increase of 0.1 to 0.2 units in the Lifestyle Advice group compared to the Standard Care group; such small differences of this magnitude are not considered to be clinically meaningful. Similarly, for log-transformed outcomes (HDL, insulin, adiponectin, leptin, NEFA and triglycerides), the estimated ratio of geometric means was very close to 1 in all cases, with the range of plausible differences indicated by the 95% CI likewise smaller than would be clinically significant (less than 10% decrease or increase in the Lifestyle Advice group compared with the Standard Care group).

**Table 2:** Cardiometabolic markers across pregnancy between randomised treatment groups

Outcome	Lifestyle intervention group	Standard care group	Estimated effect (95% CI) (unadjusted)	*P* value (unadjusted)	Estimated effect (95% CI) (adjusted)^a^	*P* value (adjusted)
Cholesterol (mmol/L)				0.856*		0.866*
Baseline	5.48 (1.22)	5.54 (1.22)	–0.04 (–0.17, 0.09)	0.521	–0.04 (–0.17, 0.09)	0.512
28 weeks	6.49 (1.47)	6.59 (1.47)	–0.07 (–0.20, 0.07)	0.320	–0.06 (–0.19, 0.08)	0.396
36 weeks	6.76 (1.51)	6.83 (1.55)	–0.08 (–0.21, 0.06)	0.273	–0.08 (–0.22, 0.06)	0.265
CRP^b,c^ (mg/L)				0.284*		0.338*
Baseline	6.82 (4.04, 11.34)	6.47 (3.36, 11.32)	1.09 (1.01, 1.18)	0.032	1.08 (1.01, 1.17)	0.036
28 weeks	6.40 (3.67, 10.43)	5.92 (3.19, 10.18)	1.12 (1.03, 1.21)	0.006	1.11 (1.03, 1.20)	0.006
36 weeks	5.35 (3.08, 8.85)	4.55 (2.49, 8.18)	1.15 (1.06, 1.25)	<0.001	1.14 (1.05, 1.23)	0.001
Glucose (mmol/L)				0.775*		0.690*
Baseline	4.78 (0.94)	4.76 (0.97)	0.01 (–0.10, 0.12)	0.864	0.01 (–0.10, 0.11)	0.910
28 weeks	4.89 (1.32)	4.83 (1.38)	0.06 (–0.05, 0.17)	0.299	0.07 (–0.04, 0.18)	0.227
36 weeks	5.12 (1.19)	5.08 (1.13)	0.04 (–0.08, 0.16)	0.534	0.03 (–0.08, 0.14)	0.622
HDL (mmol/L)				0.634*		0.577*
Baseline	1.43 (0.37)	1.44 (0.36)	0.00 (–0.03, 0.04)	0.976	0.00 (–0.03, 0.04)	0.938
28 weeks	1.49 (0.39)	1.48 (0.40)	0.01 (–0.03, 0.05)	0.589	0.01 (–0.02, 0.05)	0.492
36 weeks	1.37 (0.36)	1.39 (0.39)	–0.00 (–0.04, 0.03)	0.807	–0.00 (–0.04, 0.03)	0.860
Insulin^b,c^ (μIU/mL)						
28 weeks	18.92 (13.35, 27.16)	18.42 (13.57, 26.07)	1.03 (0.96, 1.10)	0.419	1.02 (0.96, 1.09)	0.471
Adiponectin^b^ (μg/mL)				0.097*		0.117*
Trial entry	8.16 (5.81, 12.15)	8.66 (6.31, 11.96)	0.99 (0.94, 1.04)	0.656	0.99 (0.94, 1.04)	0.650
28 weeks	7.46 (5.07, 10.61)	7.67 (5.60, 10.80)	0.95 (0.89, 1.00)	0.046	0.94 (0.89, 1.00)	0.034
36 weeks	7.83 (5.76, 10.83)	7.98 (5.73, 11.25)	1.00 (0.95, 1.06)	0.989	0.99 (0.94, 1.05)	0.787
Leptin^b^ (ng/mL)				0.257*		0.241*
Trial entry	52.21 (37.76, 69.66)	48.66 (35.12, 68.19)	1.04 (0.99, 1.09)	0.145	1.03 (0.99, 1.08)	0.160
28 weeks	60.87 (43.74, 79.72)	59.41 (43.07, 82.31)	1.00 (0.95, 1.05)	0.933	0.99 (0.94, 1.04)	0.759
36 weeks	54.15 (36.68, 74.20)	54.49 (36.76, 74.39)	1.02 (0.97, 1.08)	0.380	1.02 (0.97, 1.07)	0.538
NEFA^b,c^ (mmol/L)						
28 weeks	0.34 (0.21, 0.47)	0.35 (0.23, 0.47)	0.97 (0.90, 1.03)	0.316	0.96 (0.90, 1.03)	0.271
Triglycerides^b^ (mmol/L)						
28 weeks	2.15 (1.73, 2.64)	2.15 (1.69, 2.69)	1.00 (0.96, 1.03)	0.908	1.00 (0.97, 1.04)	0.941
LDL (mmol/L)						
28 weeks	4.54 (1.39)	4.65 (1.41)	0.10 (–0.04, 0.24)	0.152	0.09 (–0.05, 0.23)	0.192

^a^Adjustment for centre, parity, BMI category (stratification variables), Socio-Economic Indexes for Areas Index of Relative Socio-Economic Disadvantage quintile, smoking status, and maternal age at consent

^b^Outcomes log transformed for analysis; the descriptives presented for these outcomes are median and interquartile range. Model estimates for these outcomes have been back-transformed to the original scale, and are therefore ratios of geometric means (approximately ratios of medians) (Intervention/Routine Care)

^c^Outcomes modelled using Tobit regression due to presence of values below and/or above detectable limits

For outcomes with repeated measures, numbers represent the number of women with a measure at any of the three timepoints (hence included in the model); for outcomes with only one time point, numbers are the number of women with a measure at that time

All other outcomes, are mean and standard deviation, and model estimates are differences in means

*P values for the time-by-treatment interaction term (i.e. testing whether the effect of treatment differed by time point)

*CRP* C-reactive protein, *HDL* high-density lipoprotein cholesterol, *NEFA* non-esterified fatty acids, *LDL* low-density lipoprotein cholesterol

**Table 3:** Cord blood cardiometabolic markers between randomised treatment groups

Outcome	Lifestyle advice group	Standard care group	Estimated difference (95% CI) (unadjusted)	*P* value (unadjusted)	Estimated difference (95% CI) (adjusted)^a^	*P* value (adjusted)
Cholesterol (mmol/L)	1.79 (0.71)	1.80 (0.73)	0.01 (–0.07, 0.09)	0.858	0.01 (–0.07, 0.09)	0.853
CRP^b,c^ mg/L	0.30 (0.30, 0.30)	0.30 (0.27, 0.30)	1.16 (0.74, 1.82)	0.513	1.09 (0.69, 1.72)	0.701
Glucose^c^ (mmol/L)	3.53 (1.38)	3.53 (1.46)	0.00 (–0.16, 0.17)	0.968	–0.00 (–0.16, 0.16)	0.984
HDL (mmol/L)	0.63 (0.25)	0.63 (0.25)	–0.01 (–0.03, 0.02)	0.680	–0.01 (–0.04, 0.02)	0.548
Insulin^b,c^ (μIU/mL)	10.04 (6.22, 14.93)	9.41 (5.97, 14.75)	1.06 (0.98, 1.14)	0.142	1.07 (0.99, 1.15)	0.085
NEFA^b,c^ (mmol/L)	0.19 (0.13, 0.27)	0.19 (0.13, 0.28)	0.97 (0.91, 1.04)	0.424	0.96 (0.90, 1.03)	0.276
Triglycerides^b,c^ (mmol/L)	0.39 (0.27, 0.56)	0.40 (0.26, 0.57)	1.01 (0.94, 1.09)	0.767	0.98 (0.91, 1.05)	0.565
LDL (mmol/L)	1.06 (0.54)	1.07 (0.58)	0.01 (–0.05, 0.08)	0.689	0.02 (–0.05, 0.08)	0.639
Adiponectin (μg/mL)	22.35 (16.93, 28.61)	22.28 (16.77, 28.74)	1.00 (0.95, 1.05)	0.945	1.00 (0.94, 1.05)	0.897
Leptin (ng/mL)	13.06 (7.98, 20.95)	13.13 (7.90, 21.97)	0.98 (0.90, 1.07)	0.607	0.98 (0.90, 1.07)	0.701

^a^Adjustment for centre, parity, BMI category (stratification variables), Socio-Economic Indexes for Areas Index of Relative Socio-Economic Disadvantage quintile, smoking status, and maternal age at consent

^b^Outcomes were log-transformed; descriptives for these outcomes are median and interquartile range, and estimates are ratios of geometric means (approximately ratios of medians)

All other outcomes are mean and standard deviation, and estimates are differences of means

^c^Outcomes were modelled using Tobit regression due to presence of values above and/or below detection limits

*CRP* C-reactive protein, *HDL* high-density lipoprotein cholesterol, *NEFA* non-esterified fatty acids, *LDL* low-density lipoprotein cholesterol

The exception was maternal CRP concentrations, where statistically significant differences between treatment groups were observed at 28 and 36 weeks, with the concentrations in the Lifestyle Advice group approximately 10–15% higher than in the Standard Care group. However, the CRP concentrations were higher in the Lifestyle Advice group at baseline, and there was no evidence that the groups had a different trajectory over time. Thus, the differences observed at 28 and 36 weeks are likely due to the differences observed at baseline, and while likely reflecting a chance effect, they may reflect bias introduced by missing data or unmeasured confounding. In addition, while the difference between groups was statistically significant, the magnitude of the difference was small, representing at most an increase of 20%, where concentrations will often vary by 300%.

Results from unadjusted and adjusted analyses were similar, and there was no evidence to suggest that the intervention effect was modified by maternal BMI category (data not shown).

### Inflammatory markers

Mean concentrations in inflammatory markers across pregnancy and infant cord blood (Table 4) were similar between the two treatment groups, with no statistically or clinically significant differences identified. Across all outcomes at all time points, the estimated geometric mean ratio between treatment groups was close to 1 (no difference), with the 95% CIs spanning a range from at most a 15% decrease in the Lifestyle Advice group relative to the Standard Care group, to at most a 45% increase in the Lifestyle Advice group relative to the Standard Care group. Given the degree of variability in these measures (as can be seen in the interquartile ranges presented), these differences are not considered clinically relevant.

**Table 4:** Inflammatory markers across pregnancy and from cord blood between randomised treatment groups

Outcome	Lifestyle advice group	Standard care group	Unadjusted estimate (95% CI)	Unadjusted *P* value	Adjusted estimate (95% CI)^a^	Adjusted *P* value
GMCSF (pg/mL)				0.036*		0.016*
Baseline	1.02 (0.36, 4.41)	1.05 (0.36, 4.15)	1.00 (0.84, 1.19)	0.986	1.01 (0.85, 1.20)	0.916
28 weeks	0.99 (0.36, 4.20)	0.92 (0.32, 3.98)	1.06 (0.88, 1.26)	0.545	1.07 (0.90, 1.28)	0.444
36 weeks	0.99 (0.34, 4.06)	0.95 (0.33, 3.79)	1.00 (0.83, 1.19)	0.964	1.01 (0.84, 1.21)	0.913
Cord blood	0.44 (0.23, 0.82)	0.43 (0.24, 0.87)	0.81 (0.66, 0.98)	0.034	0.80 (0.65, 0.97)	0.026
IFN-γ (pg/mL)				0.638*		0.559*
Baseline	0.39 (0.13, 1.28)	0.35 (0.14, 1.24)	1.02 (0.86, 1.21)	0.809	1.02 (0.86, 1.20)	0.834
28 weeks	0.38 (0.12, 1.17)	0.34 (0.13, 1.08)	1.02 (0.86, 1.22)	0.801	1.02 (0.86, 1.21)	0.832
36 weeks	0.35 (0.11, 1.11)	0.30 (0.11, 1.03)	1.09 (0.91, 1.30)	0.333	1.08 (0.90, 1.29)	0.419
Cord blood	0.17 (0.07, 1.43)	0.20 (0.07, 1.29)	0.96 (0.79, 1.17)	0.684	0.93 (0.76, 1.13)	0.476
TNF-α (pg/mL)				0.600*		0.643*
Baseline	0.72 (0.10, 2.69)	0.68 (0.10, 2.53)	1.01 (0.81, 1.25)	0.950	1.00 (0.80, 1.24)	0.967
28 weeks	0.62 (0.10, 2.69)	0.57 (0.10, 2.14)	1.06 (0.85, 1.33)	0.581	1.05 (0.84, 1.32)	0.650
36 weeks	0.60 (0.10, 2.28)	0.47 (0.10, 2.08)	1.16 (0.93, 1.46)	0.195	1.15 (0.91, 1.44)	0.247
Cord blood	0.42 (0.12, 0.87)	0.40 (0.10, 0.82)	1.04 (0.81, 1.32)	0.766	1.04 (0.81, 1.33)	0.770
IL-1β (pg/mL)				0.883*		0.807*
Baseline	0.20 (0.10, 0.59)	0.19 (0.10, 0.53)	1.04 (0.88, 1.23)	0.621	1.04 (0.87, 1.23)	0.687
28 weeks	0.19 (0.10, 0.54)	0.17 (0.10, 0.54)	1.07 (0.90, 1.27)	0.456	1.06 (0.89, 1.26)	0.527
36 weeks	0.18 (0.10, 0.51)	0.14 (0.10, 0.47)	1.11 (0.93, 1.33)	0.234	1.12 (0.93, 1.34)	0.229
Cord blood	1.07 (0.39, 3.04)	0.99 (0.34, 2.76)	1.04 (0.86, 1.24)	0.696	1.02 (0.85, 1.22)	0.855
IL-2 (pg/mL)				0.055*		0.054*
Baseline	0.76 (0.16, 2.76)	0.65 (0.15, 2.61)	0.99 (0.81, 1.21)	0.926	1.00 (0.82, 1.23)	0.969
28 weeks	0.73 (0.12, 2.63)	0.50 (0.12, 2.01)	1.16 (0.94, 1.43)	0.159	1.17 (0.95, 1.45)	0.135
36 weeks	0.66 (0.13, 2.33)	0.45 (0.12, 1.82)	1.16 (0.94, 1.43)	0.179	1.17 (0.94, 1.45)	0.151
Cord blood	0.12 (0.12, 0.31)	0.12 (0.12, 0.30)	0.91 (0.71, 1.16)	0.444	0.91 (0.71, 1.16)	0.458
IL-4 (pg/mL)				0.154*		0.159*
Baseline	1.70 (0.53, 6.14)	1.66 (0.49, 4.89)	1.07 (0.89, 1.27)	0.476	1.06 (0.89, 1.27)	0.530
28 weeks	1.47 (0.47, 5.51)	1.23 (0.38, 4.38)	1.21 (1.01, 1.46)	0.040	1.20 (0.99, 1.44)	0.058
36 weeks	1.37 (0.55, 5.22)	1.26 (0.38, 4.18)	1.15 (0.95, 1.38)	0.152	1.13 (0.93, 1.36)	0.217
Cord blood	1.51 (0.50, 3.53)	1.39 (0.47, 3.70)	0.97 (0.80, 1.19)	0.797	0.96 (0.78, 1.17)	0.665
IL-5 (pg/mL)				0.446*		0.476*
Baseline	0.42 (0.18, 0.98)	0.39 (0.18, 0.96)	1.02 (0.90, 1.17)	0.726	1.05 (0.92, 1.20)	0.500
28 weeks	0.40 (0.18, 0.96)	0.41 (0.18, 0.85)	1.04 (0.91, 1.20)	0.544	1.06 (0.92, 1.22)	0.409
36 weeks	0.39 (0.18, 0.88)	0.31 (0.18, 0.68)	1.13 (0.98, 1.30)	0.088	1.15 (0.99, 1.32)	0.061
Cord blood	0.61 (0.31, 1.15)	0.55 (0.31, 1.03)	1.02 (0.88, 1.18)	0.806	1.03 (0.88, 1.19)	0.736
IL-6 (pg/mL)				0.857*		0.823*
Baseline	1.18 (0.61, 2.18)	1.14 (0.59, 2.07)	1.02 (0.91, 1.13)	0.773	1.02 (0.91, 1.14)	0.738
28 weeks	1.12 (0.63, 2.06)	1.11 (0.63, 2.03)	1.05 (0.94, 1.18)	0.403	1.05 (0.94, 1.18)	0.396
36 weeks	1.33 (0.76, 2.38)	1.26 (0.78, 2.15)	0.99 (0.88, 1.12)	0.911	0.99 (0.88, 1.12)	0.886
Cord blood	4.46 (1.86, 13.86)	4.32 (1.67, 14.10)	1.00 (0.88, 1.14)	0.943	1.00 (0.87, 1.14)	0.956
IL-8 (pg/mL)				0.183*		0.198*
Baseline	7.36 (3.96, 12.06)	7.26 (4.21, 12.22)	0.96 (0.86, 1.08)	0.497	0.96 (0.87, 1.07)	0.482
28 weeks	7.66 (4.42, 13.57)	7.50 (3.76, 12.41)	1.02 (0.91, 1.15)	0.738	1.04 (0.94, 1.16)	0.451
36 weeks	7.95 (4.70, 12.86)	7.29 (4.23, 12.07)	1.12 (0.99, 1.27)	0.061	1.10 (0.99, 1.23)	0.073
Cord blood	58.93 (28.11, 131.39)	58.25 (29.89, 132.60)	1.00 (0.88, 1.15)	0.953	0.99 (0.88, 1.12)	0.872
IL-10 (pg/mL)				0.721*		0.742*
Baseline	2.45 (1.65, 3.98)	2.42 (1.68, 4.02)	0.98 (0.91, 1.06)	0.573	0.98 (0.91, 1.06)	0.683
28 weeks	2.41 (1.60, 3.98)	2.32 (1.59, 3.75)	1.02 (0.94, 1.10)	0.664	1.02 (0.94, 1.10)	0.670
36 weeks	2.39 (1.63, 3.94)	2.36 (1.60, 3.74)	0.98 (0.91, 1.07)	0.709	0.99 (0.91, 1.07)	0.725
Cord blood	4.16 (2.68, 6.50)	4.20 (2.59, 6.83)	0.97 (0.89, 1.06)	0.544	0.97 (0.89, 1.06)	0.501

^a^Adjustment for centre, parity, BMI category (stratification variables), Socio-Economic Indexes for Areas Index of Relative Socio-Economic Disadvantage quintile, smoking status, and maternal age at consent

*P value for test of interaction between treatment group and time

Descriptives are median (interquartile range) and estimates are ratios of geometric means (Intervention/Control) with 95% confidence interval

*GMCSF* granulocyte macrophage-colony stimulating factor, *IFN-γ* interferon gamma, *TNF-α* tumour necrosis factor alpha, *IL* interleukin

Findings of the unadjusted and adjusted analyses were similar. Again, there was no evidence to suggest that the intervention effect was modified by maternal BMI category (data not shown).

## Discussion

Our findings indicate that provision of a dietary and lifestyle intervention during pregnancy for women who are overweight or obese was not associated with clinically important or statistically significant differences in a comprehensive and extensive range of maternal and infant cord blood concentrations of cardiometabolic and inflammatory markers. While mean concentrations of CRP were significantly higher among women in the Lifestyle Advice group at all time points, this is unlikely to be due to the intervention, and instead is more likely due to the significant difference at baseline. We also note that, as is usual for exploratory analyses or analyses of secondary outcomes, no correction for multiple comparisons has been made, and therefore any ‘statistically significant’ results should be cautiously interpreted in the context of all reported results.

Our randomised trial is the largest reported to date evaluating the effects of an antenatal lifestyle intervention for women who are overweight or obese during pregnancy on maternal or infant cord blood cardiometabolic or inflammatory measures, and utilised robust methodology, both of which have been limitations in the research literature to date. The sample size for the trial was based on the primary outcome, and there has, therefore, been no formal calculation of statistical power for these secondary outcomes. It is therefore possible that the lack of statistically significant differences is due to lack of statistical power to detect differences that exist. However, the sample size of this analysis of secondary outcomes involves over 1900 women, and almost 1200 infants. We estimate our sample size has power to detect clinically relevant differences of 0.15 standard deviations in each maternal, and 0.17 standard deviations in each infant cord blood, cardiometabolic and inflammatory marker (80% power; two-sided alpha 0.05), and is larger than the sample sizes in other reported studies. The lack of statistically significant findings, and the magnitude of the estimated effects, suggests that a very large sample size would be needed to detect differences due to the intervention, that these effects are likely to be of very small magnitude, and that the clinical significance of such small differences is questionable.

The issue of missing data is more complex. It is possible that women who provided a blood sample, or cord blood sample (or who provided samples at all time points) are different to those women who did not, and that such differences may be responsible for the differences (or lack thereof) observed between treatment groups. While we cannot know for certain whether this is the case, there are several factors supporting the notion that missing observations are unlikely to be systematically different from the observed data. Firstly, the characteristics of both the women and infants for whom cardiometabolic and inflammatory measures were available were similar between treatment groups, and similar to the full-randomised groups [39]. Similarly, while there was loss to follow-up for later maternal measures, with 36% of women missing data for at least one time point, we do not believe that women with incomplete cardiometabolic and inflammatory data are systematically different to those with complete data. The reasons for missing maternal and cord blood data are largely independent of treatment or participant characteristics. More than 90% of women in the LIMIT study consented to provide blood and cord blood samples. Missing maternal blood specimens reflect a range of circumstances, including inability to attend an appointment, the woman already having had routine clinical blood samples taken on the appointment day or, for a small number, assay or storage freezer malfunction. Missing cord blood samples largely reflect whether the midwife responsible for conducting the birth was able to obtain sufficient cord blood over and above that required for clinical purposes. Additionally, the statistical methodology used for analyses allows inclusion of participants with incomplete data, and assumes that data is missing at random (i.e. that the value of missing observations does not depend on missingness, conditional on observed data); adjustment for potential confounders increases the likelihood that this assumption is met.

We have previously reported a significant 18% relative risk reduction in the incidence of infants with birth weight above 4.0 [39] and 4.5 kg [42] following improvements in maternal diet (an increased consumption of fruits and vegetables, and a reduction in the percentage of dietary energy derived from saturated fats) and physical activity (equivalent to approximately 20 minutes brisk walking on most days of the week) [40]. However, these positive, but relatively modest improvements have been of insufficient magnitude to measurably alter cardiometabolic and inflammatory markers across pregnancy. We postulate that this may, to some extent, also account for the lack of observed effect on clinical pregnancy outcomes, including pre-eclampsia and gestational diabetes [39].

Our findings are in contrast to other smaller studies reported in the literature involving predominantly lean pregnant women. Habitual physical activity during early pregnancy has been associated with a reduction in total cholesterol and triglycerides [34], while moderate activity (4 times per week) has been shown to reduce plasma TNF-α [35]. Consumption of a low glycaemic index diet [36] and cholesterol lowering diets have [37] both been associated with alterations in maternal lipid profiles, although these findings are not universal, with others demonstrating no effect on markers of cardiovascular disease and glucose homeostasis [38].

Several randomised trials have evaluated the effect of dietary interventions [38, 46] and metformin [47, 48] among pregnant women who are obese, and have reported a range of cardiometabolic, and more limited inflammatory markers. While the findings of these trials are largely consistent with those we report, we have included a broader range of both cardiometabolic and inflammatory markers. Poston et al. [38] demonstrated an improvement in maternal dietary patterns and a modest (0.67 kg) difference in gestational weight gain following provision of antenatal dietary intervention, although this was not associated with changes in measures of glucose metabolism, including plasma insulin, or triglycerides and cholesterol (LDL, HDL, and VLDL) at 28 weeks gestation. In a smaller trial, McCarthy et al. [46] demonstrated no significant differences in maternal leptin, adiponectin and CRP measures at 28 weeks gestation following simple dietary advice. Syngelaki et al. [48] report no difference in fasting glucose concentrations at 28 weeks gestation following use of metformin during pregnancy, similar to those reported by Chiswick et al. [47]. While the EMPoWAR trial also demonstrated no significant differences in maternal cholesterol, HDL, LDL, triglycerides, leptin or NEFA, women who received metformin had lower concentrations of CRP and IL-6 at 36 weeks gestation [47]. No statistically significant differences were identified between the two treatment groups in cord blood glucose, insulin and CRP [47].

While there is a considerable literature describing associations between increasing BMI and alterations in the cardiometabolic and inflammatory profiles [18–25], any mediating effect of gestational weight gain is less certain. For example, increasing CRP concentrations have been reported to be associated with increased gestational weight gain, although there does not appear to be an association between weight gain and concentrations of either TNF-α or IL-6 [49]. Associations between gestational weight gain and maternal leptin concentrations also remain unclear, with some authors reporting an association with increased weight gain, particularly in the second trimester of pregnancy [50], while others do not [51]. Importantly, these relationships may reflect maternal pre-pregnancy BMI, with associations between gestational weight gain and leptin concentration evident among normal weight and overweight women, but not among obese women [52]. We did not control for maternal gestational weight gain when estimating the effect of the intervention on maternal or infant cardiometabolic and inflammatory measures, as this was determined after the time of randomisation. However, we have previously shown that gestational weight gain was very similar between women in the lifestyle advice and standard care groups [39], and we therefore consider it unlikely that gestational weight gain would have substantially influenced our findings.

The findings of our randomised trial indicate that provision of an antenatal dietary and lifestyle intervention for women who are overweight or obese was not associated with any statistically or clinically significant differences in maternal or infant cord blood cardiometabolic or inflammatory markers. Despite this, it would be worth considering potential relationships between maternal and infant cardiometabolic and inflammatory markers and clinical pregnancy outcomes, in addition to longer-term infant health and adiposity measures.

## Additional file

Additional file 1: LIMIT Cardiometabolic and Inflammatory Markers: Sample Numbers for All Outcomes. (DOCX 47 kb)
