# Supplementary material for: The effect of an antenatal lifestyle intervention in overweight and obese women on circulating cardiometabolic and inflammatory biomarkers: secondary analyses from the LIMIT randomised trial
Source: BMC Med. 2017 Feb 14;15:32. doi: 10.1186/s12916-017-0790-z (PMC5307888; doi:10.1186/s12916-017-0790-z)
Supplement: Additional file 1: — LIMIT Cardiometabolic and Inflammatory Markers: Sample Numbers for All Outcomes. (DOCX 47 kb) [file 12916_2017_790_MOESM1_ESM.docx]

LIMIT Cardiometabolic and Inflammatory Markers: Sample Numbers for All Outcomes

# Overall Numbers of Participants Included in This Analysis

| **Participants** | **Lifestyle Advice Group** | **Standard Care Group** | **Total** |
| --- | --- | --- | --- |
| Any Cardiometabolic Measure  - Baseline  - 28 Weeks  - 36 Weeks  - Cord Blood | 879 (100.00)  801 (91.13)  745 (84.76)  601 (68.37) | 854 (100.00)  766 (89.70)  706 (82.67)  582 (68.15) | 1733 (100.00)  1567 (90.42)  1451 (83.73)  1183 (68.26) |
| Any Measure at Any Time | 992 | 969 | 1961 |
| Any Maternal Measure at Any Time | 961 | 944 | 1905 |
| Any Infant Measure at Any Time | 601 | 582 | 1183 |

## Combinations of Measurements

| **Number of Time Points** | **Intervention** | **Standard Care** | **Overall** |
| --- | --- | --- | --- |
| **All Time Points**  **(All maternal time points)**  **(At least 2 maternal)** | 437  (634)  (830) | 415  (590)  (792) | 852  (1224)  (1622) |
| **One Time Point Only**   - BL - 28w - 36w - CB | 81  11  5  31 | 89  17  9  25 | 170  28  14  56 |
| **Two Time Points**   - BL + 28w - BL + 36w - BL + CB - 28w + 36w - 28w + CB - 36w + CB | 56  20  18  21  12  4 | 60  24  13  13  12  12 | 116  44  31  34  24  16 |
| **Three Time Points**   - BL, 28w, 36w - BL, 28w, CB - BL, 36w, CB - 28w, 36w, CB | 197  38  32  29 | 175  47  31  27 | 372  85  63  56 |

# Cytokine Measures

## Numbers of Participants

| **Measure** | **Lifestyle Advice Group** | **Standard Care Group** | **Total** |
| --- | --- | --- | --- |
| Any Cytokine Measure  - Baseline  - 28 Weeks  - 36 Weeks  - Cord Blood | 858  784  736  574 | 834  743  699  546 | 1692  1527  1435  1120 |
| GMCSF  - Baseline  - 28 Weeks  - 36 Weeks  - Cord Blood | 825  751  704  533 | 808  717  676  507 | 1633  1468  1380  1040 |
| IFNg  - Baseline  - 28 Weeks  - 36 Weeks  - Cord Blood | 787  724  669  505 | 782  688  650  481 | 1569  1412  1319  986 |
| TNFa  - Baseline  - 28 Weeks  - 36 Weeks  - Cord Blood | 820  749  699  551 | 801  709  674  524 | 1621  1458  1373  1075 |
| IL1b  - Baseline  - 28 Weeks  - 36 Weeks  - Cord Blood | 787  724  669  505 | 782  688  650  481 | 1569  1412  1319  986 |
| IL2  - Baseline  - 28 Weeks  - 36 Weeks  - Cord Blood | 803  729  683  506 | 793  696  664  481 | 1596  1425  1347  987 |
| IL4  - Baseline  - 28 Weeks  - 36 Weeks  - Cord Blood | 802  739  694  534 | 791  692  663  513 | 1593  1431  1357  1047 |
| IL5  - Baseline  - 28 Weeks  - 36 Weeks  - Cord Blood | 796  725  689  522 | 770  679  651  502 | 1566  1404  1340  1024 |
| IL6  - Baseline  - 28 Weeks  - 36 Weeks  - Cord Blood | 858  783  736  569 | 834  743  699  543 | 1692  1526  1435  1112 |
| IL8  - Baseline  - 28 Weeks  - 36 Weeks  - Cord Blood | 857  784  736  574 | 834  743  699  546 | 1691  1527  1435  1120 |
| IL10  - Baseline  - 28 Weeks  - 36 Weeks  - Cord Blood | 856  783  735  574 | 831  740  697  546 | 1687  1523  1432  1120 |

## Values Outside Detection Thresholds

| **Measure** | **Below (Lifestyle Advice)** | **Above (Lifestyle Advice)** | **Below (Standard Care)** | **Above (Standard Care)** | **Below (Total)** | **Above (Total)** |
| --- | --- | --- | --- | --- | --- | --- |
| GMCSF  - Baseline  - 28 Weeks  - 36 Weeks  - Cord Blood | 110 (13.33)  104 (13.85)  96 (13.64)  106 (19.89) | 1 (0.12)  2 (0.27)  1 (0.14)  0 (0.00) | 97 (12.00)  107 (14.92)  92 (13.61)  86 (16.96) | 3 (0.37)  4 (0.56)  4 (0.59)  0 (0.00) | 207 (12.68)  211 (14.37)  188 (13.62)  192 (18.46) | 4 (0.24)  6 (0.41)  5 (0.36)  0 (0.00) |
| IFNg  - Baseline  - 28 Weeks  - 36 Weeks  - Cord Blood | 111 (14.10)  105 (14.50)  104 (15.55)  139 (27.52) | 3 (0.38)  2 (0.28)  2 (0.30)  0 (0.00) | 116 (14.83)  99 (14.39)  116 (17.85)  133 (27.65) | 0 (0.00)  0 (0.00)  0 (0.00)  0 (0.00) | 227 (14.47)  204 (14.45)  220 (16.68)  272 (27.59) | 3 (0.19)  2 (0.14)  2 (0.15)  0 (0.00) |
| TNFa  - Baseline  - 28 Weeks  - 36 Weeks  - Cord Blood | 210 (25.61)  199 (26.57)  182 (26.04)  132 (23.96) | 7 (0.85)  5 (0.67)  6 (0.86)  0 (0.00) | 205 (25.59)  199 (28.07)  215 (31.90)  137 (26.15) | 1 (0.12)  1 (0.14)  3 (0.45)  0 (0.00) | 415 (25.60)  398 (27.30)  397 (28.91)  269 (25.02) | 8 (0.49)  6 (0.41)  9 (0.66)  0 (0.00) |
| IL1b  - Baseline  - 28 Weeks  - 36 Weeks  - Cord Blood | 111 (14.10)  105 (14.50)  104 (15.55)  139 (27.52) | 3 (0.38)  2 (0.28)  2 (0.30)  0 (0.00) | 116 (14.83)  99 (14.39)  116 (17.85)  133 (27.65) | 0 (0.00)  0 (0.00)  0 (0.00)  0 (0.00) | 227 (14.47)  204 (14.45)  220 (16.68)  272 (27.59) | 3 (0.19)  2 (0.14)  2 (0.15)  0 (0.00) |
| IL2  - Baseline  - 28 Weeks  - 36 Weeks  - Cord Blood | 191 (23.79)  187 (25.65)  165 (24.16)  271 (53.56) | 2 (0.25)  4 (0.55)  2 (0.29)  0 (0.00) | 183 (23.08)  207 (29.74)  189 (28.46)  262 (54.47) | 1 (0.13)  1 (0.14)  0 (0.00)  0 (0.00) | 374 (23.43)  394 (27.65)  354 (26.28)  533 (54.00) | 3 (0.19)  5 (0.35)  2 (0.15)  0 (0.00) |
| IL4  - Baseline  - 28 Weeks  - 36 Weeks  - Cord Blood | 105 (13.09)  88 (11.91)  89 (12.82)  59 (11.05) | 7 (0.87)  6 (0.81)  4 (0.58)  0 (0.00) | 94 (11.88)  112 (16.18)  108 (16.29)  52 (10.14) | 2 (0.25)  2 (0.29)  3 (0.45)  0 (0.00) | 199 (12.49)  200 (13.98)  197 (14.52)  111 (10.60) | 9 (0.56)  8 (0.56)  7 (0.52)  0 (0.00) |
| IL5  - Baseline  - 28 Weeks  - 36 Weeks  - Cord Blood | 202 (25.38)  178 (24.55)  195 (28.30)  52 (9.96) | n/a | 204 (26.49)  182 (26.80)  210 (32.26)  58 (11.55) | n/a | 406 (25.93)  360 (25.64)  405 (30.22)  110 (10.74) | n/a |
| IL6  - Baseline  - 28 Weeks  - 36 Weeks  - Cord Blood | 10 (1.17)  6 (0.77)  2 (0.27)  0 (0.00) | 3 (0.35)  5 (0.64)  2 (0.27)  20 (3.51) | 4 (0.48)  4 (0.54)  4 (0.57)  0 (0.00) | 4 (0.48)  2 (0.27)  2 (0.29)  20 (3.68) | 14 (0.83)  10 (0.66)  6 (0.42)  0 (0.00) | 7 (0.41)  7 (0.46)  4 (0.28)  40 (3.60) |
| IL8  - Baseline  - 28 Weeks  - 36 Weeks  - Cord Blood | 60 (7.00)  47 (5.99)  30 (4.08)  2 (0.35) | 2 (0.23)  5 (0.64)  2 (0.27)  55 (9.58) | 55 (6.59)  42 (5.65)  38 (5.44)  5 (0.92) | 2 (0.24)  5 (0.67)  0 (0.00)  64 (11.72) | 115 (6.80)  89 (5.83)  68 (4.74)  7 (0.63) | 4 (0.24)  10 (0.65)  2 (0.14)  119 (10.63) |
| IL10  - Baseline  - 28 Weeks  - 36 Weeks  - Cord Blood | 4 (0.47)  0 (0.00)  3 (0.41)  0 (0.00) | n/a | 1 (0.12)  2 (0.27)  1 (0.14)  0 (0.00) | n/a | 5 (0.30)  2 (0.13)  4 (0.28)  0 (0.00) | n/a |

# Other Biological Measures

## Numbers of Participants

| **Measure** | **Lifestyle Advice Group** | **Standard Care Group** | **Total** |
| --- | --- | --- | --- |
| **Any Metabolic Marker**  - Baseline  - 28 Weeks  - 36 Weeks  - Cord Blood | 879  801  745  601 | 854  766  706  582 | 1733  1567  1451  1183 |
| **Cholesterol**  - Baseline  - 28 Weeks  - 36 Weeks  - Cord Blood | 876  800  743  595 | 851  762  704  574 | 1727  1562  1447  1169 |
| **CRP**  - Baseline  - 28 Weeks  - 36 Weeks  - Cord Blood | 876  799  742  400 | 851  763  704  375 | 1727  1562  1446  775 |
| **Glucose**  - Baseline  - 28 Weeks  - 36 Weeks  - Cord Blood | 876  800  743  585 | 851  763  704  560 | 1727  1563  1447  1145 |
| **HDL**  - Baseline  - 28 Weeks  - 36 Weeks  - Cord Blood | 875  800  742  592 | 850  763  703  571 | 1725  1563  1445  1163 |
| **Insulin**  - 28 Weeks (fasting)  - Cord Blood | 567  595 | 545  575 | 1112  1170 |
| **NEFA**  - 28 Weeks  - Cord Blood | 795  584 | 761  565 | 1556  1149 |
| **Triglycerides**  - 28 Weeks  - Cord Blood | 800  592 | 762  569 | 1562  1161 |
| **LDL**  - 28 Weeks  - Cord Blood | 800  581 | 761  554 | 1561  1135 |
| **Adiponectin**  - Baseline  - 28 Weeks  - 36 Weeks  - Cord Blood | 748  604  608  536 | 723  568  588  516 | 1471  1172  1196  1052 |
| **Leptin**  - Baseline  - 28 Weeks  - 36 Weeks  - Cord Blood | 748  604  608  535 | 723  568  588  516 | 1471  1172  1196  1051 |

## Values Outside Detection Thresholds

| **Measure** | **Below (Lifestyle Advice)** | **Above (Lifestyle Advice)** | **Below (Standard Care)** | **Above (Standard Care)** | **Below (Total)** | **Above (Total)** |
| --- | --- | --- | --- | --- | --- | --- |
| **Cholesterol**: n/a (no values outside detection limits) | | | | | | |
| **CRP**  - Baseline  - 28 Weeks  - 36 Weeks  - Cord Blood | 0 (0.00)  0 (0.00)  0 (0.00)  189 (47.25) | n/a | 1 (0.12)  0 (0.00)  0 (0.00)  158 (42.13) | n/a | 1 (0.06)  0 (0.00)  0 (0.00)  347 (44.77) | n/a |
| **Glucose**  - Baseline  - 28 Weeks  - 36 Weeks  - Cord Blood | 0 (0.00)  0 (0.00)  0 (0.00)  1 (0.17) | n/a | 0 (0.00)  0 (0.00)  0 (0.00)  1 (0.18) | n/a | 0 (0.00)  0 (0.00)  0 (0.00)  2 (0.17) | n/a |
| **HDL**: n/a (no values outside detection limits) | | | | | | |
| **Insulin**  - 28 Weeks  - Cord Blood | 0 (0.00)  2 (0.34) | 2 (0.35)  0 (0.00) | 0 (0.00)  1 (0.17) | 1 (0.18)  0 (0.00) | 0 (0.00)  3 (0.26) | 3 (0.27)  0 (0.00) |
| **NEFA**  - 28 Weeks  - Cord Blood | 7 (0.88)  10 (1.71) | 0 (0.00)  0 (0.00) | 8 (1.05)  6 (1.06) | 1 (0.13)  0 (0.00) | 15 (0.96)  16 (1.39) | 1 (0.06)  0 (0.00) |
| **Triglycerides**  - 28 Weeks  - Cord Blood | 0 (0.00)  8 (1.35) | n/a | 0 (0.00)  12 (2.11) | n/a | 0 (0.00)  20 (1.72) | n/a |
| **LDL**: n/a (derived variable, set to missing if any constituent values were outside limits) | | | | | | |
| **Adiponectin**  - Baseline  - 28 Weeks  - 36 Weeks  - Cord Blood | 1 (0.13)  0 (0.00)  0 (0.00)  0 (0.00) | n/a | 1 (0.14)  0 (0.00)  0 (0.00)  0 (0.00) | n/a | 2 (0.14)  0 (0.00)  0 (0.00)  0 (0.00) | n/a |
| **Leptin**: n/a (no out of range values) | | | | | | |
